# Efficiency limits for photoelectrochemical water-splitting

**DOI:** 10.1038/ncomms13706

**Published:** 2016-12-02

**Authors:** Katherine T. Fountaine, Hans Joachim Lewerenz, Harry A. Atwater

**Affiliations:** 1 NG Next, 1 Space Park Drive, Redondo Beach, California 90278, USA; 2 Deparment of Chemistry and Chemical Engineering, California Institute of Technology, 1200 East California Boulevard, Pasadena, California 91125, USA; 3 Division of Engineering and Applied Sciences, California Institute of Technology, 1200 East California Boulevard, Pasadena, California 91125, USA; 4 Joint Center for Artificial Photosynthesis, California Institute of Technology, 1200 East California Boulevard, Pasadena, California 91125, USA

## Abstract

Theoretical limiting efficiencies have a critical role in determining technological viability and expectations for device prototypes, as evidenced by the photovoltaics community's focus on detailed balance. However, due to their multicomponent nature, photoelectrochemical devices do not have an equivalent analogue to detailed balance, and reported theoretical efficiency limits vary depending on the assumptions made. Here we introduce a unified framework for photoelectrochemical device performance through which all previous limiting efficiencies can be understood and contextualized. Ideal and experimentally realistic limiting efficiencies are presented, and then generalized using five representative parameters—semiconductor absorption fraction, external radiative efficiency, series resistance, shunt resistance and catalytic exchange current density—to account for imperfect light absorption, charge transport and catalysis. Finally, we discuss the origin of deviations between the limits discussed herein and reported water-splitting efficiencies. This analysis provides insight into the primary factors that determine device performance and a powerful handle to improve device efficiency.

## Abstract (web summary)

Theoretical limiting efficiencies play a critical role in determining technological viability and expectations for device prototypes. Here, the authors present a unified framework for photoelectrochemical device performance through which previous limiting efficiencies can be understood and contextualized.

In the photovoltaics community, the detailed balance limit serves as a gold standard to which all device efficiencies are compared^1^. The seminal paper written by Shockley and Queisser in 1961 presents photovoltaic limiting efficiencies as a function of a single parameter, the semiconductor bandgap, under the assumption that the only loss mechanism is radiative recombination in the semiconductor. While many corollaries to this limit exist^2^,^3^,^4^,^5^,^6^,^7^,^8^,^9^, the transcendence of this analysis is enabled by its elegance, analytic simplicity and basis in the ultimate limit of semiconductor device physics.

Due to their complex, multicomponent nature, an equivalent analogue to the detailed balance limit does not exist for photoelectrochemical (PEC) devices. A number of articles have been written on the limiting efficiencies of photoelectrochemical devices^3^,^10^,^11^,^12^,^13^,^14^,^15^, each with slightly different approaches and assumptions to arrive at different limiting efficiency values. In this article, we aim to present a unified framework for photoelectrochemical device performance through which all previous limiting efficiencies can be understood. To do so, we first present the analytic equations and solutions for the limiting efficiencies of photoelectrochemical water-splitting devices based on the ultimate limits of device physics; limiting efficiencies reported here are consistent with those presented in references^3^,^13^,^15^. Subsequently, we examine the validity of these ideal limits and consider more realistic limits based on existing materials, similar to those presented in references^10^,^11^,^12^,^14^; the realistic limits are presented and discussed as a function of five parameters: semiconductor absorption fraction, semiconductor external radiative efficiency (ERE), series resistance, shunt resistance and catalytic exchange current density. These five parameters directly correlate with the three governing physical phenomena of photoelectrochemical device operation—light absorption (absorption fraction), charge carrier transport (ERE, series resistance and shunt resistance), catalysis (catalytic exchange current density); the parameter variation study demonstrates the varying impact of each phenomenon on overall device efficiency and efficiency limits. Finally, this analysis is contextualized via a comparison of the discussed limits with reported water-splitting efficiencies.

## Results

### Outline

The following analysis of water-splitting device efficiencies is divided into five parts. First, we derive the analytic equation that governs a variable-junction photoelectrochemical device and its efficiency. This set of equations is generally applicable to any photoelectrochemical device. Second, we present the absolute limiting efficiencies for a photoelectrochemical device for water-splitting for both single and dual junction photodiode units. Subsequently, we present two sets of realistic limiting efficiencies based on currently available high performance (real_1_) and Earth abundant (real_2_) materials. Next, we consider the effects of five representative parameters on the limiting efficiency and the corresponding semiconductor bandgap(s). Finally, we contextualize this theoretical efficiency analysis via a brief discussion of reported efficiencies. This analysis provides insight into the primary factors currently limiting device efficiency and guidance to researchers on (1) the most powerful handles to improve device efficiency and (2) routes to maximize device efficiency for a given set of material and device parameters.

### Analytic equation for PEC device operation and efficiency

In previous work, we derived analytic equations for a variable-junction photoelectrochemical device^16^,^17^. This derivation is briefly summarized below. The characteristic current-voltage relationship for a photoelectrochemical device lends itself to an inverse formulation, *V*_PEC_(*j*), where *V*_PEC_ is the voltage generated by the device that is available for conversion into chemical energy, *j* is the device current density, *V*_PV,*i*_(*j*) is the inverse current-voltage relationship for the *i*th photodiode component, *V*_cat,a_(*j*) and *V*_cat,c_(*j*) are the current-dependent overpotentials of the anodic and cathodic catalysts, respectively, *V*_series_ is the series resistance due to electrolyte transport through solution and membrane, formulated as *jR*_series_, and *E*_rxn_ is the electrochemical potential of the desired chemical reaction.


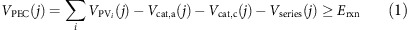


The photodiode voltage is described by an inverse formulation of the diode equation, where *j*_0_ is the reverse saturation (dark) current, *n*_d_ is the ideality factor, *k*_B_ is the Boltzmann constant and *T* is the device temperature.


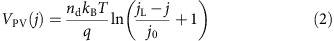


Butler-Volmer kinetics are selected to describe the current density-dependent catalytic overpotentials, *V*_cat_(*j*); this model and the further simplified Tafel equation are commonly used to fit electrocatalyst behaviour, however, it should be noted that Butler-Volmer kinetics are only accurate for outer sphere single electron transfer reactions^18^. Specifically, we employ an inverse formulation of the Butler-Volmer equation, found by assuming that the charge transfer coefficients of a specific catalyst in the forward and reverse directions, *α*_f_ and *α*_r_, are equal, where *R* is the universal gas constant, *n*_e_ is the number of electrons associated with the reaction, *F* is Faraday's constant and *j*_0,cat_ is the catalytic exchange current density. A comparison of this formulation, the more standard Tafel equation and the complete Butler-Volmer equation is provided in Supplementary Note 1 and Supplementary Fig. 1, as well as in previous work^17^.


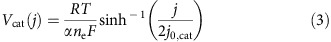


The reaction proceeds when the photoelectrochemical device voltage is greater than or equal to the electrochemical potential required to drive the reaction, *E*_rxn_, as defined in equation (1). The maximum efficiency occurs when the voltage is precisely equal to the required electrochemical potential because this maximizes the device current density; this point is defined as the device operating point, *V*_op_(*j*_op_)=*E*_rxn_ (see Supplementary Note 2 and Supplementary Fig. 2 for visualization). The device operating current density is directly proportional to the device efficiency, *η*_PEC_, according to the following equation, where *f*_FE_ is the Faradaic efficiency and *P*_in_ is the incident solar power.


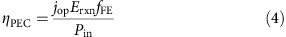


### Absolute limiting efficiencies

To determine the absolute limiting efficiencies of single and dual junction photoelectrochemical devices for water-splitting, the following assumptions were made (and are also summarized in Table 1):

1. Illumination with the AM1.5G spectrum:

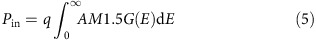


2. Complete absorption of all photons above the bandgap of the semiconductor.

3. A detailed balance model for the photodiode dark current, assuming only radiative recombination in the semiconductor:


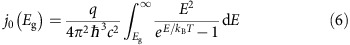


This equation assumes a perfect antireflective coating and perfect back reflector. For the dual junction calculations, the dark current of the upper junction is multiplied by a factor of 2 to account for emission from the upper and lower surfaces. A detailed treatment of angular emission probability, more formally known as the etendue, can be found in Markvart *et al*.^19^

4. Diode ideality factor, *n*_d_, of 1.

5. Catalytic overpotentials are assumed to be negligible. Mathematically, this assumption corresponds to infinite catalytic exchange current densities. This condition can be approached by (1) discovery of new catalysts with very high-catalytic exchange current densities and (2) high-surface area catalyst and high catalyst loading, which increases the effective catalytic exchange current density when normalized to device area.

6. Charge transfer coefficients of 0.5.

7. No series resistance.

8. The electrochemical potential for water-splitting at standard conditions, *E*_rxn_=1.23 V.

9. Unity Faradaic efficiency.

The analysis is restricted to single and dual junctions because additional junctions do not result in any efficiency gains. In fact, the maximum ideal triple junction efficiency for water-splitting is 28.3%, which is significantly lower than the maximum dual junction efficiency (40.0%). This drop in efficiency with increasing junction number (beyond 2) is contrary to photovoltaic efficiencies and occurs because additional photovoltage beyond that required to kinetically split water does not increase efficiency; furthermore, the increased number of current-matched junctions reduces device photocurrent, which directly lowers efficiency, as shown in equation 4.

**Figure 1:**
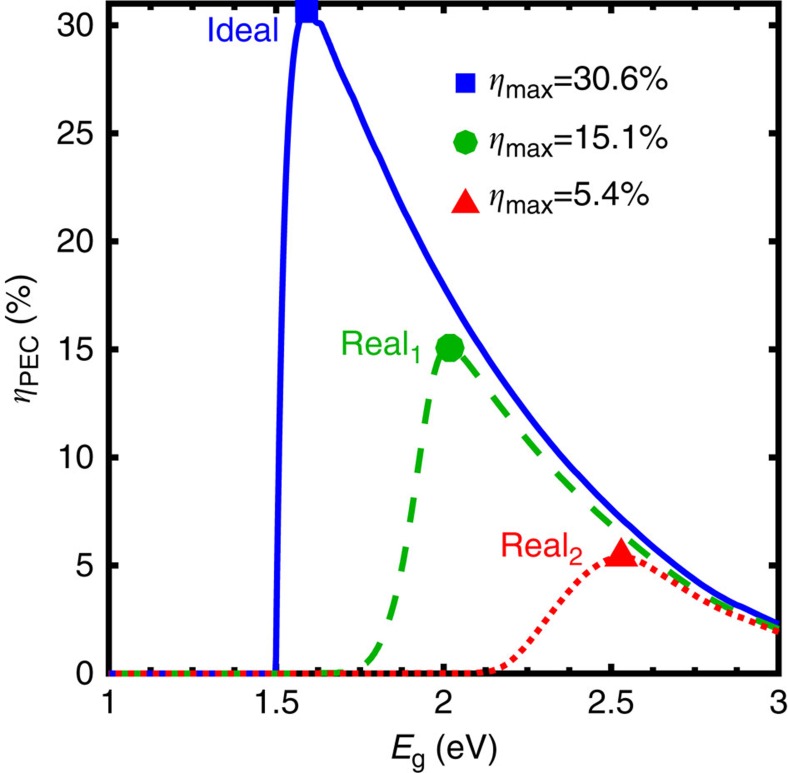
Single junction limiting efficiencies. Limiting efficiencies (*η*_PEC_) versus semiconductor bandgap (*E*_g_) for ideal case (blue solid line, *η*_max_=30.6%, *E*_g_=1.59 eV), high-performance realistic case (green dashed line, *η*_max_=15.1%, *E*_g_=2.05 eV) and Earth abundant realistic case (red dotted line, *η*_max_=5.4%, *E*_g_=2.53 eV); parameter values used for each case are tabulated in Table 1.

**Figure 2:**
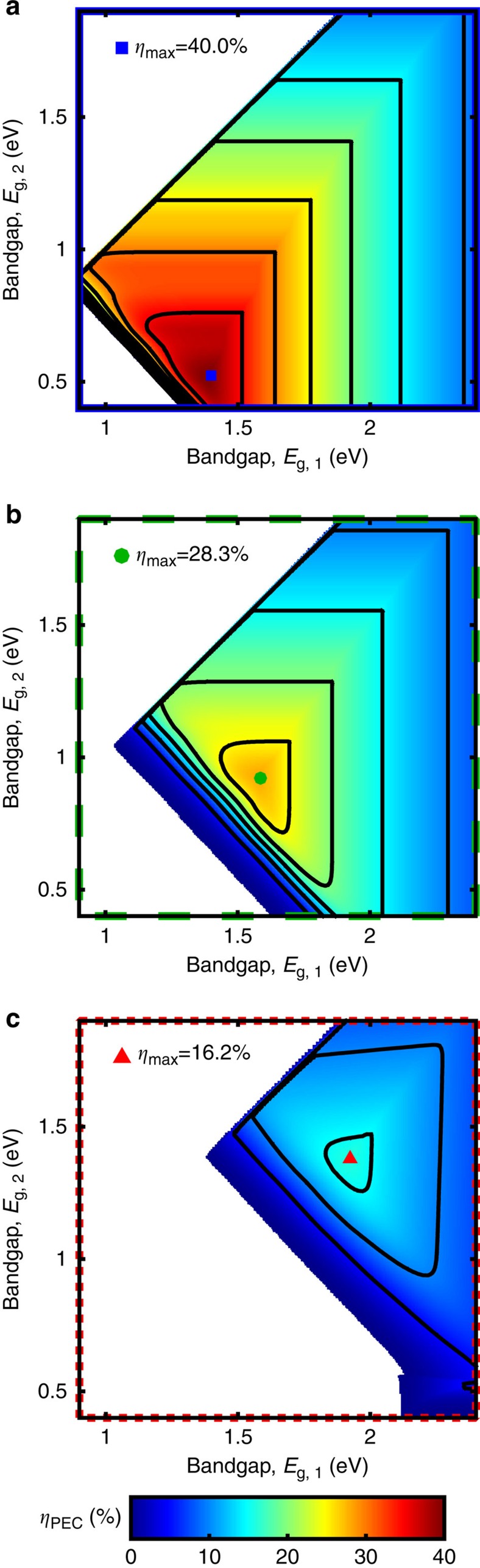
Dual junction limiting efficiencies. Limiting efficiencies (*η*_PEC_) versus semiconductor bandgaps (*E*_g_) for (**a**) ideal case (*η*_max_=40.0%, *E*_g_=1.40, 0.52 eV), (**b**) high-performance realistic case (*η*_max_=28.3%, *E*_g_=1.59, 0.92 eV) and (**c**) Earth abundant realistic case (*η*_max_=16.2%, *E*_g_=1.93, 1.38 eV), where contour lines mark every 5% and maximum efficiency points are indicated; a constant colour scale is used for **a**–**c**; parameter values used for each case are tabulated in Table 1.

Figure 1 (blue solid line) and Fig. 2a display the limiting efficiencies as a function of semiconductor bandgap for single and dual junction photoelectrochemical devices, respectively, that result from this ideal set of assumptions. The maximum single and dual junction efficiencies are 30.6% at a bandgap of 1.59 eV, and 40.0% with bandgaps of 0.52 and 1.40 eV, respectively. The single junction efficiency trend, Fig. 1(blue solid line), strongly resembles that of the single junction photovoltaic detailed balance limit, with a few notable differences. The efficiency decreases for bandgaps smaller and larger than the maximum efficiency point due to the inverse correlation between light absorption and voltage generation. The efficiency decreases with increasing bandgap due to decreased absorption of the incident solar spectrum, but the photoelectrochemical efficiency decreases more rapidly than the photovoltaic efficiency because the voltage converted to chemical energy remains constant (*E*_rxn_) despite the larger photovoltages supplied by wide bandgap semiconductors. According to equation (4), the decrease in efficiency is directly proportional to the decrease in photocurrent. For small bandgaps, the photoelectrochemical efficiency drops off sharply to zero, in contrast with the gradual decay of photovoltaic efficiency with decreasing bandgap; this sharp cutoff in efficiency occurs due to insufficient photovoltage to drive the reaction. Another critical difference is that the maximum single junction photoelectrochemical device efficiency is about 3% lower than that of a single junction photovoltaic device, despite the fact that this calculation has neglected any catalyst- or solution-related losses. This difference exists because the output voltage of a photovoltaic device is variable, whereas the required output voltage of a water-splitting device is fixed at 1.23 V. The result of this requirement is that the maximum efficiency occurs for the semiconductor with a sufficient bandgap to generate 1.23 V of photovoltage, which is a bandgap of 1.59 eV assuming detailed balance.

The ideal dual junction efficiency contour plot (Fig. 2a) exhibits a similar trend, but in two dimensions; it has a sharp turn-on of efficiency for bandgaps just large enough to supply the water-splitting efficiency voltage and a gradual decline of efficiency beyond the peak due to decreasing light absorption. The dual junction water-splitting efficiency falls short of the dual junction photovoltaic efficiency for the same reason: fixed photovoltage.

### Realistic limiting efficiencies

The absolute limiting efficiencies presented in the previous section represent theoretical limits for an ideally constructed device with ideal photodiodes and catalysts. This section presents realistic limiting efficiencies based on material and device parameters reported in literature for (1) high performance (real_1_) and (2) Earth abundant photodiodes and catalysts (real_2_). Five material parameters capture non-ideal photodiode and catalyst performance—semiconductor absorption fraction, *f*_abs_, semiconductor ERE, series resistance, *R*_S_, shunt resistance, *R*_Sh_ and catalytic exchange current density, *j*_0,cat_. Absorption fraction and catalytic exchange current density represent the efficiency of light absorption and catalysis. The efficiency of charge carrier transport is divided into three parameters, where ERE represents intrinsic semiconductor material quality in accordance with the photovoltaic community^20^, and series and shunt resistance represent device fabrication quality. ERE serves as a straightforward, linear modification to the detailed balance limit (as shown in assumption #2, below). In the detailed balance limit, the only loss mechanism is radiative recombination, as required by black body emission of the semiconductor above its band edge. This limit implies that a photodiode at open circuit voltage will re-emit all photons that were absorbed. This ideal case corresponds to an ERE of 1. In real absorbers, non-radiative recombination mechanisms exist, so that the re-emitted photons at open circuit voltage are only some fraction of those absorbed; this fraction is the ERE. Additional details and tabulated values for photovoltaic materials can be found in ref. 20. For semiconductor–liquid junctions, the ERE factor can also be treated as a simple dark current modification factor (see equation 8) to align the open circuit voltage of the model to the built-in voltage of the semiconductor-electrolyte interface^18^,^21^. The series and shunt resistance terms account for non-idealities in charge transport that are not captured by the ERE parameter and primarily affect the device fill factor. Series resistance reduces the fill factor and, at high values, also the short circuit current density; sources of series resistance include solution resistance to electrolyte transport, interfacial resistance at the semiconductor|catalyst interface, and resistance to majority carrier flow in the semiconductor. Shunt resistance pathways lower the fill factor and, at significantly low values of shunt resistance, can also reduce open circuit voltage; shunt resistance arises from partial shorting of diode junctions, which can occur quite readily in semiconductor—liquid junctions due to the ease with which liquid electrolyte can intercalate into pinholes in the semiconductor^22^,^23^,^24^.

### High-performance realistic efficiencies

We first consider realistic limiting efficiencies for high-performance materials and devices. In these calculations, we include the effects of non-ideal light absorption, ERE and catalytic exchange current density, but neglect the effects of series and shunt resistances because fill factors of current high-performance photodiodes are approaching their ideal values^25^. Specifically, the following modifications to the ideal efficiency calculations in the previous section were made (also summarized in Table 1):

1. Absorption of 90% of incident photons above the bandgap of the semiconductor. Reflection and incomplete and parasitic absorption by catalyst materials or other device components contribute to this 10% loss. Analytically, we express this non-unity absorption fraction, *f*_abs_, as a modification to the ideal diode equation:

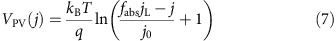


2. An ERE of 3%, meaning that the detailed balance radiative recombination represents 3% of the total (radiative and non-radiative) recombination, and thus 3% of the total dark current. This value is characteristic of high-performance III–V materials^20^. Analytically, ERE modifies the ideal photodiode dark current:


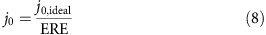


3. Catalytic exchange current densities of 1 mA cm^−2^ and 10^−3^ mA cm^−2^ for the hydrogen (cathodic) and oxygen (anodic) evolution reactions, respectively. These values are consistent with the best reported values in literature for Pt and IrO_2 _(refs 26, 27).

This analysis is limited to single and dual junction devices because triple junction maximum efficiencies are lower than that of dual junctions under these assumptions (25.4 versus 28.3%); although, the gap between dual and triple junction device efficiencies has narrowed in comparison to the ideal case.

Figure 1 (green dotted line) and Fig. 2b display the limiting efficiencies as a function of semiconductor bandgap for single and dual junction photoelectrochemical devices, respectively, under these assumptions. An identical colour scale is used for each contour plot in Fig. 2 for facile visual comparison. The maximum efficiency is 15.1% for a single junction device with a bandgap of 2.05 eV, and is 28.3% for a dual junction device with bandgaps of 0.92 and 1.59 eV. The high-performance realistic efficiency plots exhibit similar trends to the ideal efficiency plots, but the efficiencies are significantly lower and the optimum bandgaps are substantially higher. Due to the series addition of photovoltages, the inclusion of realistic experimental values in this calculation does not affect the dual junction device efficiency as dramatically as the single junction device efficiency. All three of the modifications stated above have a role in lowering the efficiency and increasing the optimum bandgaps, but as discussed in greater detail in the parameter variation section, the introduction of finite catalytic exchange current densities has the most dramatic effect.

### Earth abundant realistic efficiencies

Next, we consider realistic limiting efficiencies for Earth abundant materials and devices that currently have less than optimal performance characteristics. In these calculations, we include the effects of all five parameters—non-ideal light absorption, ERE, series resistance, shunt resistance and catalytic exchange current density. Specifically, the following modifications to the ideal efficiency calculations in the previous section were made (and are also summarized in Table 1):

1. Absorption of 90% of incident photons above semiconductor bandgap.
2. An ERE of 10^−6^, consistent with reported values for Earth abundant materials^20^.
3. Catalytic exchange current densities of 1 mA cm^−2^ and 10^−5^ mA cm^−2^ for the hydrogen (cathodic) and oxygen (anodic) evolution reactions, respectively. These values are consistent with reported values for Earth abundant catalysts in literature, such as NiMo for hydrogen evolution, and NiZn, CoFe and NiMoFe for oxygen evolution^28^.
4. Normalized series and shunt resistance values of 0.1 and 10, respectively, which each result in approximately a 10% reduction in fill factor. Series and shunt resistances are normalized to the characteristic resistance of an ideal photodiode, which is the ratio of the open circuit voltage to the short circuit current density. This normalization results in an approximately bandgap-independent reduction in fill factor. A more detailed explanation and discussion of these parameters can be found in Supplementary Note 3. Analytically, incorporation of the shunt and series resistance terms results in a transcendental equation for the photodiode current-voltage equation, where *R*_S_ and *R*_Sh_ are the absolute (non-normalized) series and shunt resistances, respectively:

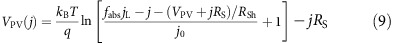


This analysis includes single, dual and triple junction devices because the additional assumed reductions in material and device parameters lead to a maximum triple junction maximum efficiency that exceeds that of a dual junction (17.3 versus 16.2%).

**Figure 3:**
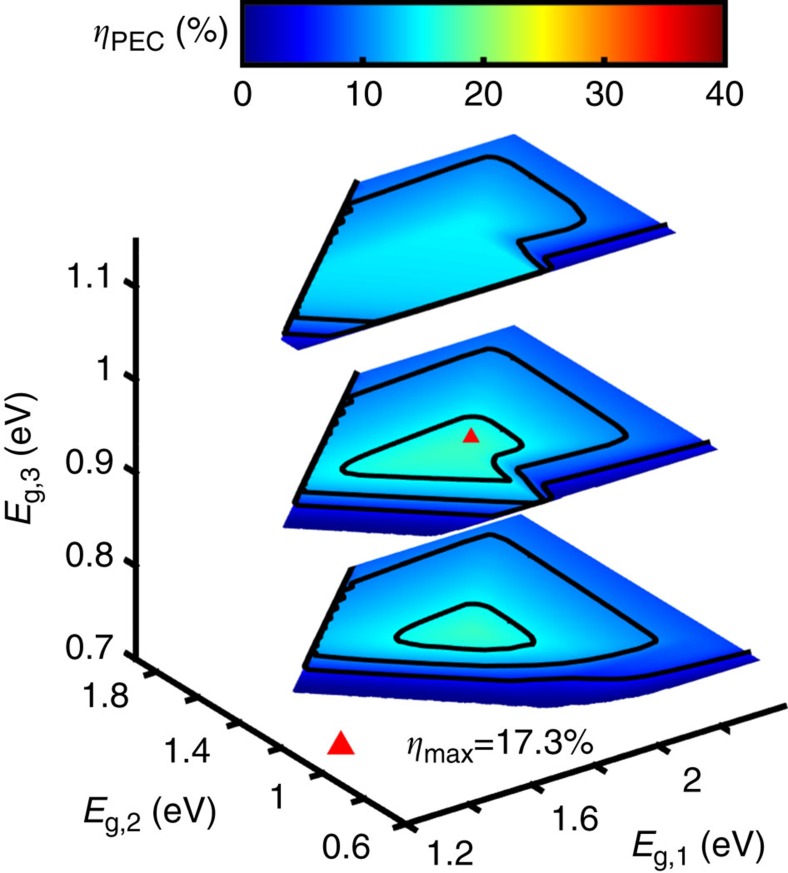
Triple junction limiting efficiencies. Limiting efficiencies (*η*_PEC_) versus upper and middle semiconductor bandgaps (*E*_g,1_, *E*_g,2_) for the Earth abundant realistic case at three lower bandgap values (*E*_g,3_=0.73, 0.93 and 1.13), where the middle plane displays the maximum triple junction efficiency under these conditions (parameter values are tabulated in Table 1); contour lines mark every 5% and the maximum efficiency point is indicated (*η*_max_=17.3%, *E*_g_=1.91, 1.36, 0.93 eV); colour scale matches that used in Fig. 2.

Figure 1(red dotted line), Fig. 2c, and Fig. 3 display the Earth abundant limiting efficiencies as a function of semiconductor bandgap for single, dual and triple junction photoelectrochemical devices that result from the above specified set of assumptions. Figure 3 displays the triple junction efficiency as a function of its upper two bandgaps for three values of the lower bandgap (0.73, 0.93 and 1.13 eV), and uses a colour scale identical to that of Fig. 2 for facile value comparison. The maximum efficiency is 5.4% for a single junction device with a bandgap of 2.53 eV, 16.2% for a dual junction device with bandgaps of 1.38 and 1.93 eV, and 17.3% for a triple junction device with bandgaps of 1.91, 1.36 and 0.93 eV (contained in the middle contour of Fig. 3). As expected, the Earth abundant realistic (real_2_) efficiency plots also exhibit similar trends to the ideal efficiency and high-performance realistic (real_1_) efficiency plots, but efficiencies are significantly lower and the optimum bandgaps are substantially higher; the dual and triple junction devices are also affected significantly less than the single junction devices by non-ideal charge carrier transport. Additionally, the inclusion of series and shunt resistance terms that affect the fill factor result in a softening of the efficiency ‘turn-on' with increasing bandgap, which is most evident in the single junction device trend (Fig. 1, red dotted line). It is also important to note that the maximum triple junction device efficiency exceeds that of a dual junction device due to non-ideal charge carrier transport and large catalyst overpotentials, indicating that use of Earth abundant materials may (presently) necessitate the use of three or more junctions to maximize device efficiency.

### Effect of parameter variation on device efficiency

**Figure 4:**
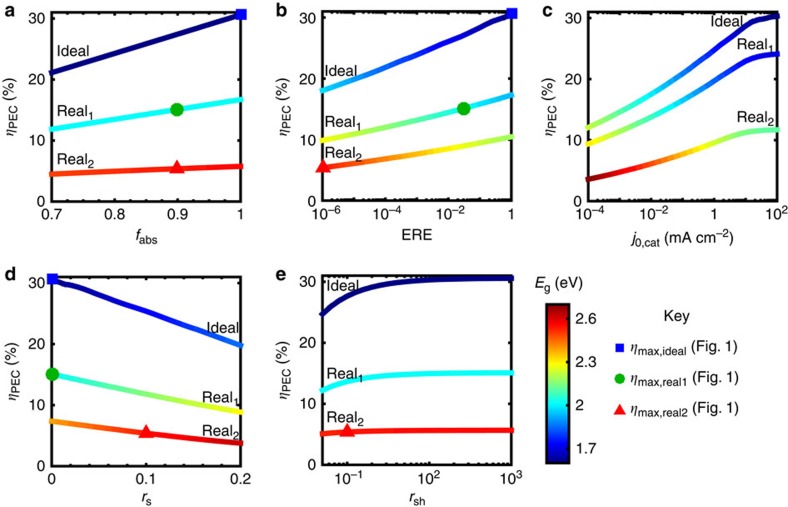
Single junction device efficiency parameter dependence. Limiting efficiencies (*η*_max_) for each single junction case (see Table 1) as a function of a single parameter variation–(**a**) absorption fraction (*f*_abs_), (**b**) external radiative efficiency (ERE), (**c**) catalytic exchange current density (*j*_0,cat_ in mA cm^−2^), (**d**) normalized series resistance (*r*_s_), and (**e**) normalized shunt resistance (*r*_sh_); the colour variation indicates the semiconductor bandgap corresponding to the maximum device efficiency; the blue squares, green circles and red triangles indicate the position of the maximum efficiencies for the ideal, high-performance realistic and Earth abundant realistic cases shown in Fig. 1.

**Figure 5:**
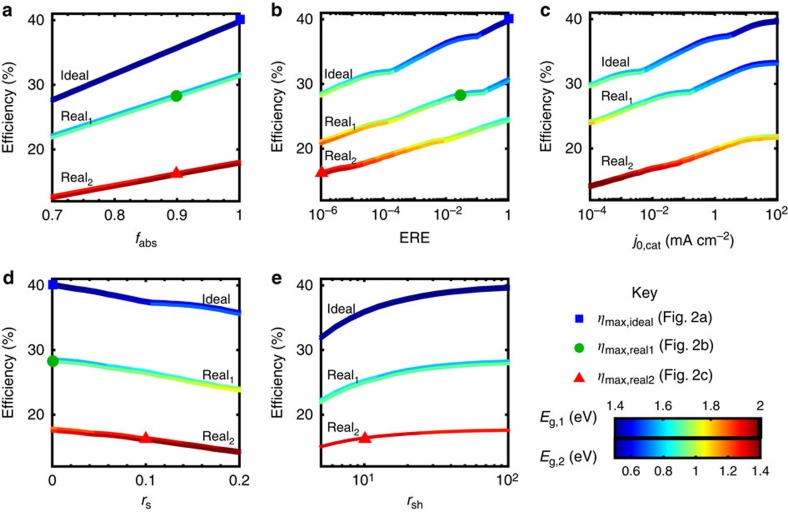
Dual junction device efficiency parameter dependence. Limiting efficiencies (*η*_max_) for each dual junction case (see Table 1) as a function of a single parameter variation–(**a**) absorption fraction (*f*_abs_), (**b**) external radiative efficiency (ERE), (**c**) catalytic exchange current density (*j*_0,cat_ in mA cm^−2^), (**d**) normalized series resistance (*r*_s_) and (**e**) normalized shunt resistance (*r*_sh_); the dual colour band of the line indicates the two semiconductor bandgaps (upper bandgap material over lower bandgap material) corresponding to the maximum device efficiency; the blue squares, green circles and red triangles indicate the position of the maximum efficiencies for the ideal, high-performance realistic and Earth abundant realistic cases shown in Fig. 2.

A detailed analysis of the dependence of device efficiency on key parameters provides insight into the most powerful handles to improve device performance. To this end, we present the dependence of the maximum achievable device efficiency and the corresponding semiconductor bandgap(s) on the aforementioned parameters for single (Fig. 4) and dual (Fig. 5) junction devices; specifically, parameter variations are performed around the three base cases presented in the previous sections (ideal, high-performance realistic—real_1_, and Earth abundant realistic—real_2_). The absorption fraction is varied between 0.7 and 1.0 (Figs 4a and 5a), the ERE between 10^−6^ and 1 (Figs 4b and 5b), the catalytic exchange current density between 10^−4^ and 10^2^ mA cm^−2^ (Figs 4c and 5c); the normalized series resistance between 0 and 0.2 (Figs 4d and 5d), and the normalized shunt resistance between 5 and 10^3^ (Figs 4e and 5e). The maximum device efficiency for a given set of parameters is plotted on the *y* axis and colour is used to display the corresponding bandgap(s); for the dual junction devices, the coloured line is a double band, where the upper component represents the wider bandgap, *E*_g,1_, as indicated in the figure legends. The colour variation representation of the optimum bandgaps is designed to illustrate trends; for easier extraction of the precise numeric bandgap values, plots of bandgap versus parameter corresponding to Figs 4 and 5 can be found in Supplementary Figs 3, 4 and 5. The maximum efficiency points corresponding to the ideal, high-performance realistic (real_1_) and Earth abundant realistic (real_2_) cases presented in previous sections are marked on Figs 4 and 5; each point on Figs 4 and 5 originates from a calculation analogous to those in the previous sections with one modified parameter. Note that the ideal, real_1_ and real_2_ points are omitted from the *j*_0,cat_ variation plots because the anodic and cathodic exchange currents were lumped into a single parameter for simplicity. An animation is provided in Supplementary Movie 1 to visualize the connection between Fig. 3b and Fig. 1.

This sensitivity analysis reveals that the efficiency of a single junction device is predominantly controlled by catalyst performance (catalytic exchange current density—*j*_0,cat_), whereas the maximum efficiency of a dual junction device is strongly affected by the optoelectronic performance of the semiconductor photodiodes in addition to catalyst performance (all five parameters—*j*_0,cat_, *f*_abs_, ERE, *r*_s_ and *r*_sh_).

For single junction devices, variation of the catalytic exchange current density around all three cases (ideal, real_1_, real_2_) results in the largest modulation in device efficiency and semiconductor bandgap (Fig. 4c). As the catalytic exchange current density decreases and, thus, kinetic overpotential increases, the semiconductor bandgap required to drive the water-splitting reaction increases, which leads to a precipitous drop in efficiency due to reduced solar spectrum conversion, as illustrated in Fig. 1 and previously discussed. Series resistance (Fig. 4d) also has a distinct effect on single junction device efficiency because, even at moderate values, series resistance shifts the maximum power point to lower voltages and thus significantly lowers the photocurrent near the maximum power point, thereby lowering the efficiency for a given bandgap and pushing the bandgap for maximum efficiency to higher values. Variation of the ERE (Fig. 4b), absorption fraction (Fig. 4a) and shunt resistance (Fig. 4e) have significantly less influence than catalytic exchange current density and series resistance on the single device efficiency. Unlike series resistance, shunt resistance primarily lowers the photocurrent, but, at moderate values, does not significantly shift the voltage at maximum power; therefore, shunt resistance lowers the efficiency due to photocurrent, but the effect is more moderate because the optimum bandgap remains relatively constant. The dependence of device efficiency on absorption fraction is almost linear, and the bandgap corresponding to maximum efficiency is nearly unaffected due to the weak logarithmic dependence of photovoltage on photocurrent (equations (2) and (7)). The ERE exhibits a similar, nearly log-linear correlation with device efficiency; however, the semiconductor bandgap and device efficiency are more strongly correlated with ERE than with absorption fraction because a lower ERE translates to a lower photovoltage and, therefore, a large bandgap semiconductor is required to generate an equivalent photovoltage. The optimum bandgap trends provide guidance to experimentalists for (i) initial semiconductor selection in device design given known achievable material parameters, and (ii) when a re-design of their device would be beneficial to its performance. For instance, a significant improvement in catalytic exchange current density significantly shifts the optimum bandgap for maximum device efficiency and may suggest the use of a different semiconductor material with a lower bandgap whereas improvements in semiconductor absorption do not.

Unlike single junction devices, all five parameters have a significant effect on the overall dual junction device efficiency (Fig. 5). The effect of poor catalyst performance on dual junction device efficiency is mitigated due to the series addition of two photovoltages. Similar to the single junction device, an increase in photovoltage is needed to compensate for the increase in kinetic overpotential, but in a dual junction device, this photovoltage increase is split between two semiconductors, which translates to a smaller increase in required bandgaps and, consequently, less overall effect on device efficiency. Conversely, variation of the charge transport parameters (ERE, series resistance and shunt resistance) and absorption fraction affect the performance of each semiconductor individually and, therefore, the effect of these four parameters on overall efficiency is largely the same across single and dual junction devices. The kinks in the efficiency curves for ERE and catalyst exchange current density variations are a direct product of the AM1.5G spectrum; dips in atmospheric transparency translate to quick jumps in the ideal semiconductor bandgap, and thus, the efficiency. These kinks are not observed in all curves because the ideal bandgaps do not cross these values.

### Theoretical limits versus reported efficiencies

To place the above theoretical analysis into context, this final section contains a brief consideration of discrepancies between theoretical and experimental efficiencies, based on three exemplary water-splitting devices—(1) a minimally integrated, dual junction Pt_black_|Si|AlGaAs|RuO_2_ device with 18.3% efficiency from Licht *et al*. in 2000 (ref. 29); (2) an integrated dual junction Rh|GaInP|GaInAs|RuO_2_ device with 14% efficiency from May *et al*. in 2015 (ref. 30); and (3) a triple junction Co-Pi|BVO|a-Si|nc-Si|Pt device with 5.2% efficiency from Han *et al*. in 2014 (ref. 31). A recent review paper from Ager *et al*.^32^ provides a more comprehensive summary of record water-splitting efficiencies as a function of time and device subtype. These three devices were selected because they mimic our three cases (ideal, high performance and Earth abundant) and highlight the varying limiting factors in different device design strategies. In the device by Licht *et al*., a high efficiency, dual junction photodiode component is wired to large surface area, high-performance catalyst components. The device by May *et al*. also uses a high-performance dual junction photodiode, but is monolithically integrated with a high-performance catalyst and fully immersed in solution. The device by Han *et al*. takes a very different tact by employing all Earth abundant materials and sacrificing performance.

Despite the use of high-performance materials and large catalyst areas, the 18.3% efficiency device by Licht *et al*., recognized as the current record water-splitting efficiency by Ager *et al*. as of February 2015, falls far short of the ideal and high-performance realistic (real_1_) limiting efficiencies (40.0% and 28.3%, respectively). The primary reason for this efficiency gap is non-ideal bandgap selection. The ideal (real_1_) efficiency limits for the bandgap combinations in the Licht device, 1.1 and 1.6 eV, and the May device, 1.26 and 1.78 eV, are 27.2% (24.5%) and 22.8% (20.5%), respectively. The ideal (real_2_) efficiency limit for the bandgap combinations in the Han device, 2.4, 1.7, and 1.1 eV, is 9.2% (4.2%). The integration of ideal bandgap materials for multijunction devices is a challenge that also faces the photovoltaics community. For high-performance materials, the principal issue is that high-quality material, which translates to high ERE, requires lattice-matched materials, and the lattice-matching requirement restricts the available bandgap combinations^33^. Lattice-mismatched multijunction photovoltaics are the subject of much ongoing research, including strategies such as inverted metamorphic and pseudomorphic designs, to name a few, and progress in this area will be instrumental for water-spitting device efficiency improvement^34^,^35^. In the realm of Earth abundant materials, the challenge is identification and optimization of a material with the appropriate bandgap, and for liquid junctions in the photoelectrochemistry community, also the appropriate band alignment and stability. When their non-ideal bandgap combinations are taken into account, both the Licht and the May devices are within roughly 6% of the adjusted high-performance realistic (real_1_) efficiency limits; and the Han device is within 4% of the ideal limit and actually exceeds the Earth abundant realistic (real_2_) limit, clearly illustrating the primary limitation in Earth abundant devices—photoelectrode bandgap.

The factors accounting for the remaining 6% discrepancy between realized and theoretical efficiency are different for the Licht and May devices. The Licht device uses large area electrodes consisting of high-performance catalysts that are positioned in such a way as to not interact with light incident on the photodiode unit. Therefore, the majority of the losses in this device are due to less than ideal photodiode performance. An examination of the J–V curve of their photodiode reveals that both incomplete light absorption, non-ideal EREs and shunt and series resistances have a role in the reduced efficiency; most notably, their fill factor is only 77% (∼10% below the ideal). The high-performance real efficiency limit trend with shunt resistance (Fig. 5e) shows that a 10% drop in fill factor (to *r*_sh_ of 10) results in a ∼5% drop in maximum efficiency; this value agrees well with the observation that the Licht device operating current was ∼5% below their short circuit current and well-aligned with their maximum power point. The May device faces different, and additional challenges due to the direct integration of catalyst on the photodiode surface. As illustrated in the supplementary information of May *et al*. and discussed theoretically in ref. 16, the loading of catalyst on the light incident side of the device has a tradeoff^30^; low catalyst loading results in slow catalyst turn-on and high-catalyst loading blocks light transmission. As a result, the May device loses significant current density (∼4 mA cm^−2^) due to parasitic catalyst light absorption. May *et al*. also cite surface resistance and recombination at the semiconductor catalyst interface as sources of loss, indicating non-ideal EREs and series resistance. Conversely, the primary factor limiting the Han device is the BVO performance; despite the use of gradient W-doping, the high resistivity of BVO drastically reduces the device fill factor, and thus, its efficiency. Ultimately, these three exemplary devices illustrate that there is room for improvement both in individual component performance, particularly for Earth abundant materials, as well as their integration into water-splitting devices.

## Discussion

In summary, we presented limiting efficiencies of water-splitting photoelectrochemical devices under ideal and realistic conditions, *and* arbitrary intermediate conditions through parameter variation studies. We first defined the general analytic equation that governs the current-voltage characteristic of a variable-junction photoelectrochemical device and its efficiency. Second, we presented the limiting efficiencies (both ideal and experimentally realistic) of a water-splitting photoelectrochemical device for both single and dual junction photodiode units. Subsequently, we considered the effects of five parameters—semiconductor absorption fraction, semiconductor ERE, series resistance, shunt resistance and catalytic exchange current density—on the limiting efficiency and the corresponding semiconductor bandgap(s) and, thus, illustrated the varying impacts of the three main phenomena (light absorption, charge carrier transport and catalysis) on device performance. Finally, we contextualize this theoretical efficiency analysis by examining reported experimental results for three exemplary water-splitting devices. This analysis provides a framework through which one can understand all previous reported limiting efficiencies with various assumed values and also provides insight into the primary factors limiting device performance and the most powerful handles to improve device efficiency.

### Data availability

The AM1.5G spectrum data used for the efficiency calculations was derived from the public domain resource, NREL-RREDC: http://rredc.nrel.gov/solar/spectra/am1.5/. Additional data that support the findings of this study, including source code, are available from the corresponding author upon request.

## Additional information

**How to cite this article:** Fountaine, K. T. *et al*. Efficiency limits for photoelectrochemical water-splitting. *Nat. Commun.* **7,** 13706 doi: 10.1038/ncomms13706 (2016).

**Publisher's note**: Springer Nature remains neutral with regard to jurisdictional claims in published maps and institutional affiliations.

## Supplementary Material

Supplementary Information Supplementary Figures 1-5, Supplementary Notes 1-3 and Supplementary References.

Supplementary Movie 1 Evolution of the single junction limiting efficiency vs. bandgap as a function of ERE. Left panel contains the ideal line from Fig. 4b of the main text and right panel is Fig. 1 (blue line) of main text for varying values of ERE (evolves in time from 10-6 to 1). Right panel marker indicates maximum efficiency value at a given ERE, which corresponds to the point plotted (and marked) on the left panel.

## Figures and Tables

**Table 1:**
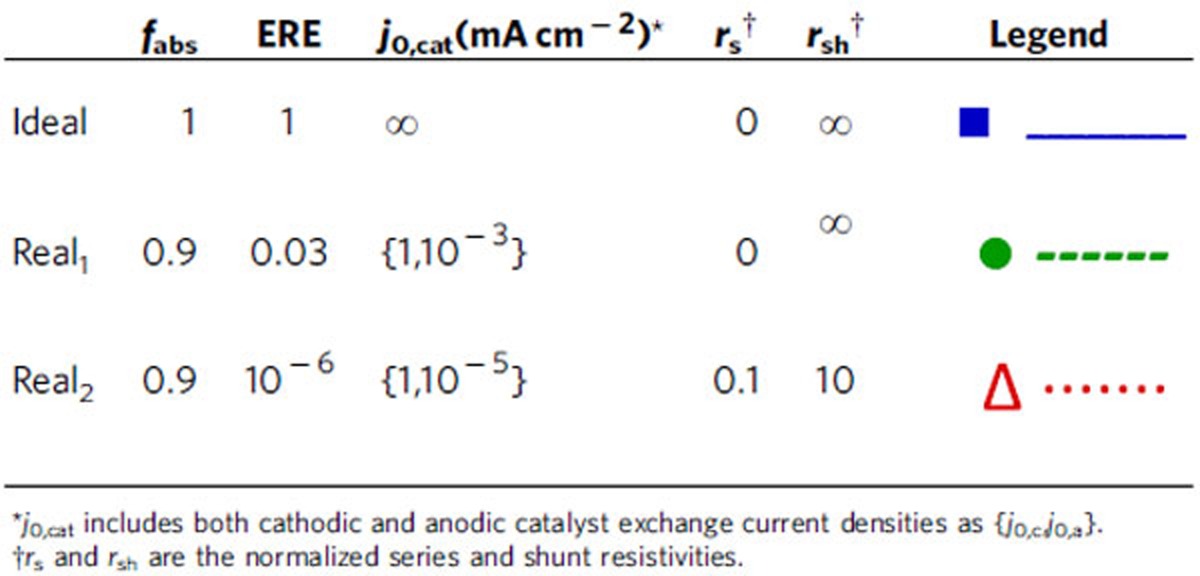
Selected parameter values for the three limiting efficiency cases.
