# Supplementary material for: Efficiency limits for photoelectrochemical water-splitting
Source: Nat Commun. 2016 Dec 2;7:13706. doi: 10.1038/ncomms13706 (PMC5146289; doi:10.1038/ncomms13706)
Supplement: Supplementary Information — Supplementary Figures 1-5, Supplementary Notes 1-3 and Supplementary References. [file ncomms13706-s1.pdf]

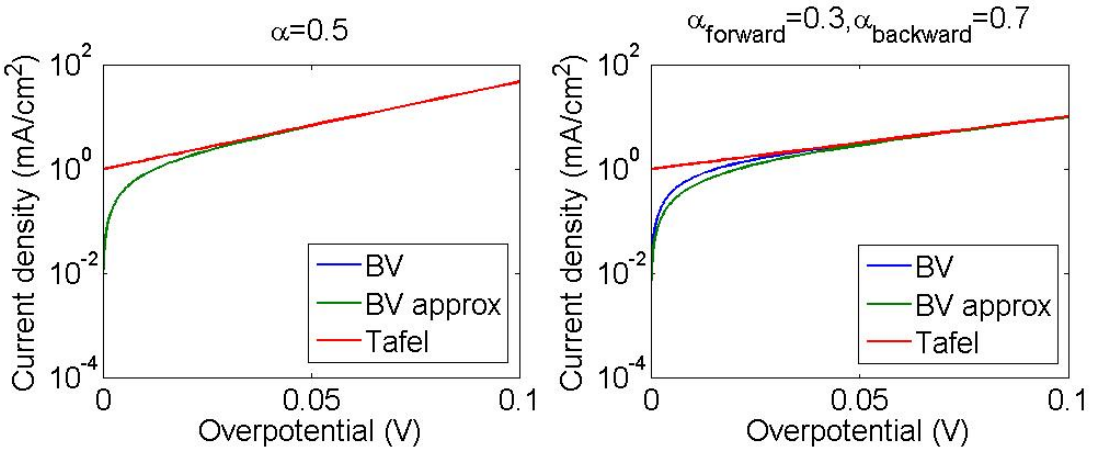

Supplementary Figure 1: Comparison of kinetic approximations. *Comparison of current density vs. overpotential curves for full Butler-Volmer kinetics, the sinh approximation for Butler-Volmer kinetics used in the text, and the Tafel equation, under (a) equal forward and backward charge transfer coefficients,α=0.5 and (b) unequal charge transfer coefficients,* $\alpha_{forward}$*=0.5 and* $\alpha_{backward}$*=0.7.*

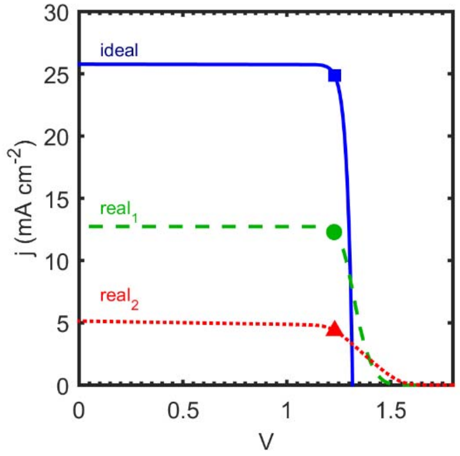

Supplementary Figure 2: Operating curves. *J-V curves for the single junction photoelectrochemical devices that maximize device efficiency for the three cases discussed in the text (ideal, high performance realistic, and earth abundant realistic); shapes mark the operating point at 1.23V (the water splitting reaction potential).*

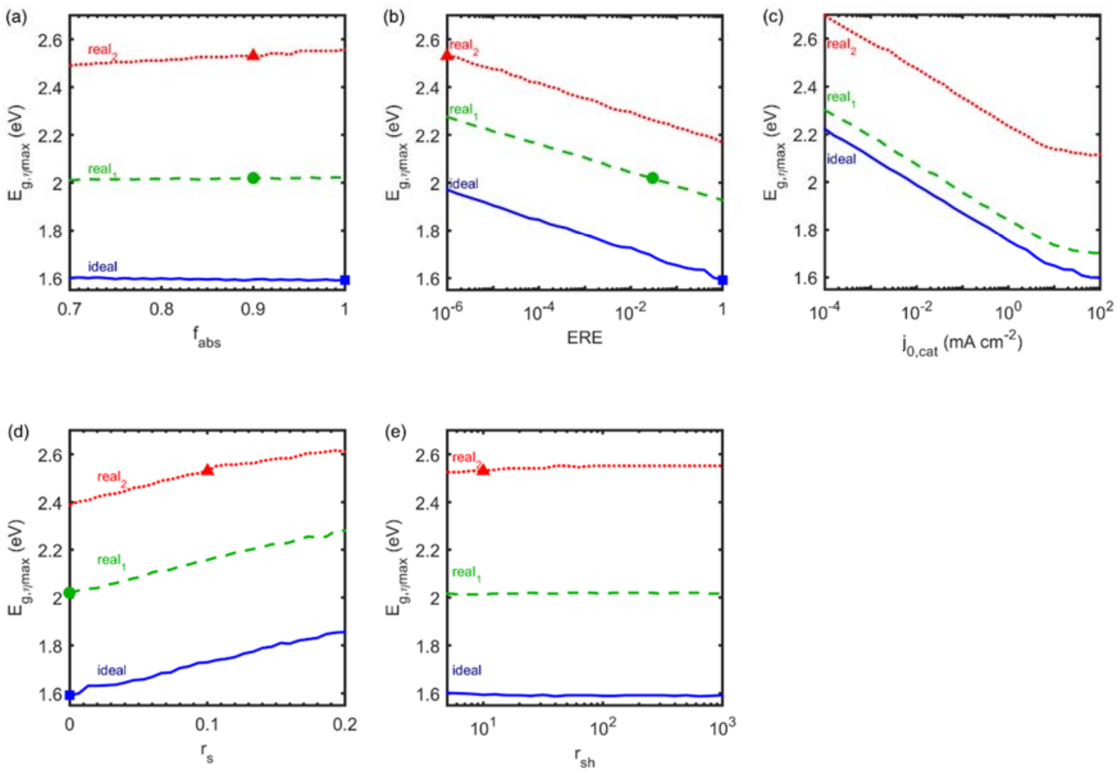

Supplementary Figure 3: *Optimum bandgap trends with parameter for single junction devices, corresponding to the color scale of Figure 4 in the main text.*

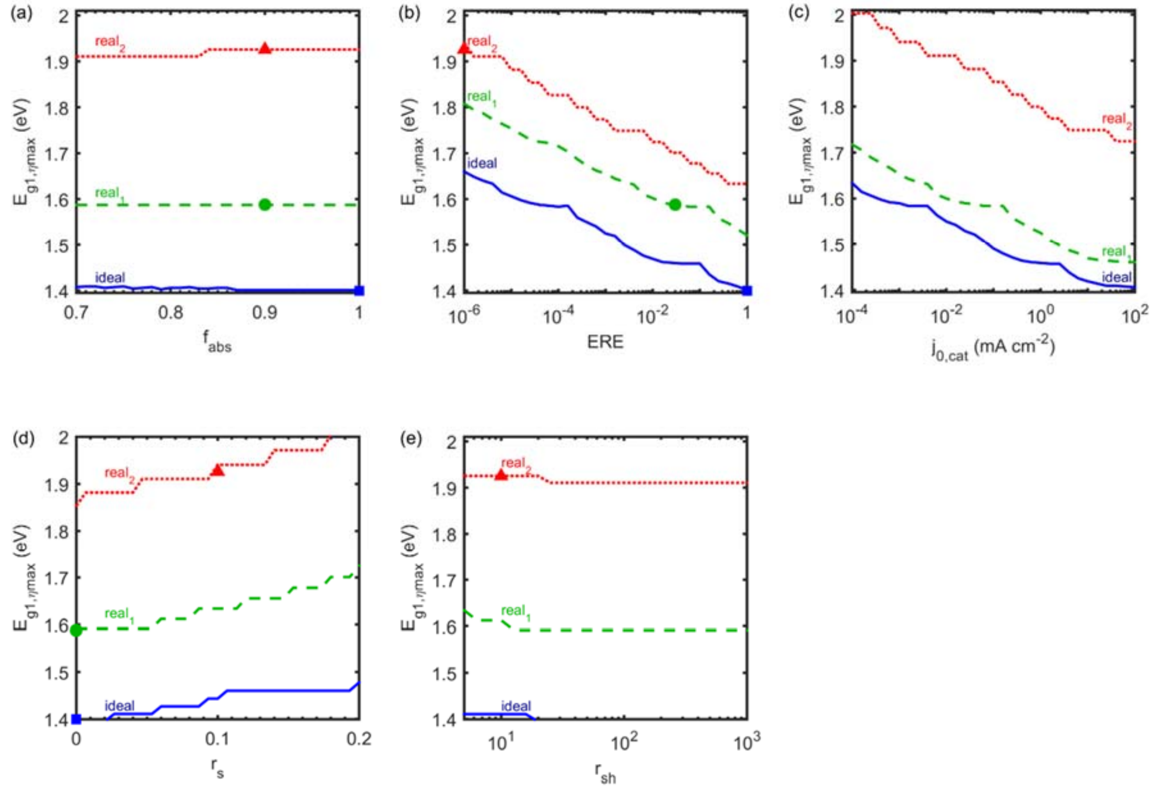

Supplementary Figure 4: *Optimum upper bandgap trends with parameter for dual junction devices, corresponding to the color scale of Figure 5 in the main text*

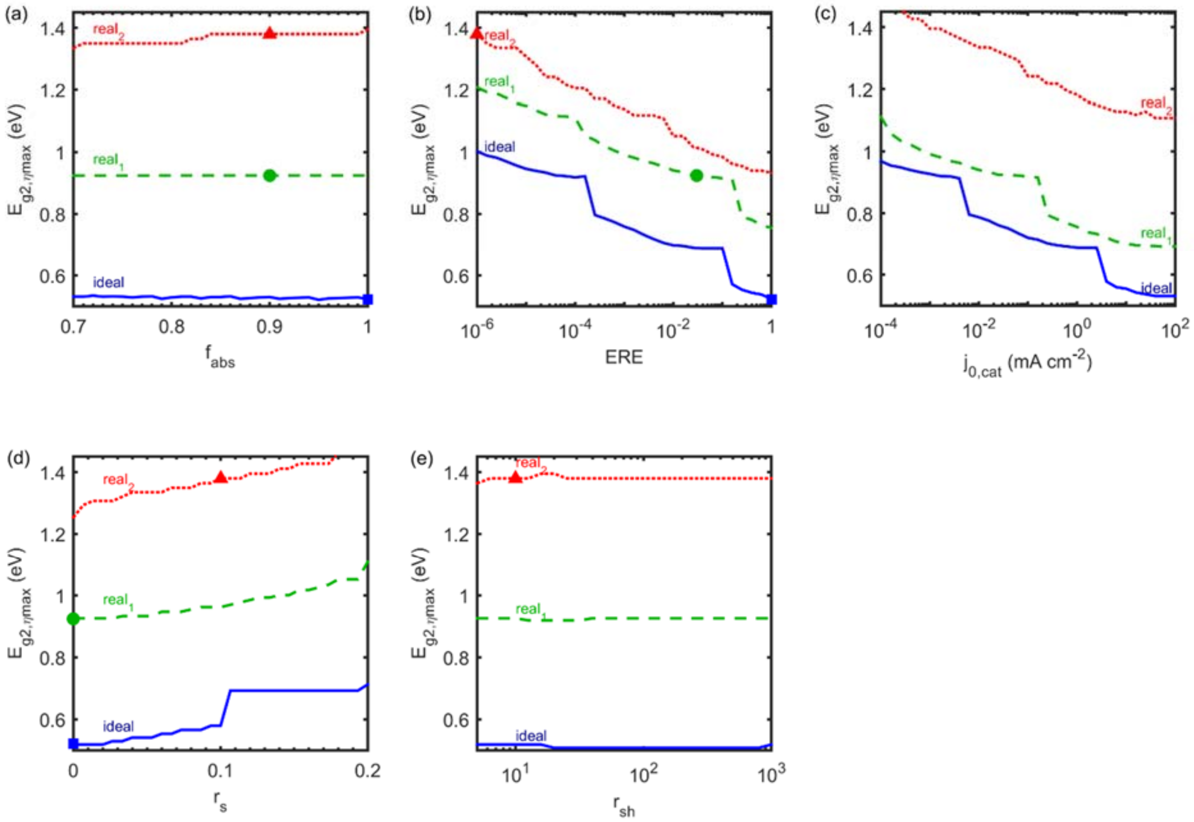

Supplementary Figure 5: *Optimum lower bandgap trends with parameter for dual junction devices, corresponding to the color scale of Figure 5 in the main text.*

Supplementary Note 1. Evaluation of kinetic approximation

In the text, an inverse *J-V* formula for kinetics is found from Butler-Volmer kinetics, by assuming equal forward and backward charge transfer coefficients of 0.5. This approximation is used in favor of the Tafel equation due to its ability to more closely match kinetic behavior at low overpotentials, which is particularly important for high performance catalysts and devices with low current densities. To demonstrate this, we present here a comparison of the three different J-V formulas:

$$j_{\mathrm{BV}}(V_{\mathrm{cat}}) = j_0\left(e^{\alpha_{\mathrm{forward}} n_{\mathrm{e}} F V_{\mathrm{cat}}/RT} - e^{-\alpha_{\mathrm{backward}} n_{\mathrm{e}} F V_{\mathrm{cat}}/RT}\right) \tag{1}$$

$$j_{\mathrm{BV,approx}}(V_{\mathrm{cat}}) = 2 j_0 \sinh\left(\frac{\alpha n_{\mathrm{e}} F V_{\mathrm{cat}}}{RT}\right) \tag{2}$$

$$j_{\mathrm{Tafel}}(V_{\mathrm{cat}}) = j_0 e^{\alpha n_{\mathrm{e}} F V_{\mathrm{cat}}/RT} \tag{3}$$

Supplementary Figure 1 displays these results for a catalyst with an exchange current density of 1 $mA\cdot cm^{-2}$, under two different conditions- (a) where the forward and backward charge transfer coefficients are equal and set to 0.5 and (b) where the forward and backward charge transfer coefficients are unequal and set to 0.3 and 0.7, respectively. In the first case (Supplementary Figure 1a), the Butler-Volmer equation (Eqn. (1), Supp. Fig. 1a blue line) and the Butler Volmer approximation (Eqn. (2), Supp. Fig. 1a green line) are identical because the trigonometric identity used is completely accurate when the two exponential factors are equal. In the second case (Supplementary Figure 1b), the identity is not completely accurate because the two exponential factors are unequal and there is some error between the approximation used in the text and complete Butler-Volmer kinetics. However, in both cases, the approximation of the Butler-Volmer equation is more accurate than the Tafel equation (Supp. Fig 1, red line) at low overpotentials. The Tafel equation overestimates the overpotential and, therefore, results in an underestimation of device efficiency for high performance catalysts and/or low performance photodiodes.

Supplementary Note 2. Operating J-V curve for a photoelectrochemical device

Supplementary Figure 2 shows the *j-V* curves for the single junction devices that maximize efficiency under the ideal (blue, solid), high performance realistic (green, dashed), and earth abundant realistic (red, dotted) assumptions – the maximum efficiency points from Fig. 1 of the main text. These curves illustrate the effects of the different parameter values. The ideal *j-V* curve is an ideal photovoltaic curve, the high performance realistic curve has a slower onset due to moderate catalytic overpotentials, and the earth abundant realistic curve has a poor fill factor due to resistances and a very slow onset due to large catalyst overpotentials. The short circuit current decreases dramatically from ideal to high performance realistic to earth abundant realistic due to the increase in bandgap required to drive the reaction. The bandgap increases due to non-negligible catalyst overpotentials and loss in photovoltage due to non-unity external radiative efficiency and resistances.

Supp. Fig. 2 also illustrates the direct correspondence of device efficiency to the device operating current at the reaction potential, which is marked on each curve (blue square, green circle, red triangle) to indicate the device operating point.

Supplementary Note 3. Justification of normalized series and shunt resistance

In the main text, we use normalized series and shunt resistance values in place of absolute series and shunt resistances because the absolute resistance values have very different magnitudes of effect on small bandgap diodes and large bandgap diodes. The goal of the resistance analyses is to demonstrate the effect of device quality on PEC efficiency and PEC design; therefore, we need a parameter that affects device quality relatively uniformly across all diode bandgaps. In this section, we demonstrate that the normalized series and shunt resistances used in the text have an approximately uniform effect on diode fill factor. These derivations were adapted from pveducation.org.[1]

3.1 Series resistance

For moderate values of series resistance, R_S, in an otherwise ideal diode, the modified maximum power point, P_{MP}', can be approximated as the ideal maximum power point, $P_{MP,ideal}$, minus the series resistance power loss:

$$P_{MP}^{'} = P_{\mathrm{MP,ideal}} - j_{\mathrm{MP}}^{2} R_{\mathrm{S}} = P_{\mathrm{MP,ideal}} \left(1 - \frac{j_{\mathrm{MP,ideal}}}{V_{\mathrm{MP,ideal}}} R_{\mathrm{S}} \right) \quad (4)$$

For an ideal diode, the characteristic resistance of a photodiode, R_{Ch}, can be approximated by the ratio of the open circuit voltage, V_{OC}, and the short circuit current density, J_{SC}.[1]

$$R_{\mathrm{Ch}} = \frac{V_{\mathrm{MP}}}{j_{\mathrm{MP}}} = \frac{V_{\mathrm{OC}}}{j_{\mathrm{SC}}} \quad (5)$$

Using this approximation in conjunction with the assumption that open circuit voltage and short circuit current are unaffected by the resistance term, we arrive at a relation between the modified fill factor, the ideal fill factor and the normalized series resistance, r_s,

$$P_{\mathrm{MP}}^{'} = P_{\mathrm{MP,ideal}}\left(1-\frac{R_{\mathrm{S}}}{R_{\mathrm{Ch}}}\right) = P_{\mathrm{MP,ideal}}\left(1-r_{\mathrm{S}}\right) \tag{6}$$

$$V_{\mathrm{OC}}^{'}\, j_{\mathrm{SC}}^{'}\, ff^{'} = V_{\mathrm{OC,ideal}}\, j_{\mathrm{SC,ideal}}\, ff_{\mathrm{ideal}}\left(1-r_{\mathrm{S}}\right) \tag{7}$$

$$ff^{'} = ff_{\mathrm{ideal}}\left(1-r_{\mathrm{S}}\right) \tag{8}$$

where the normalized series resistance, r_{S}, is defined as

$$r_{\mathrm{S}} = \frac{R_{\mathrm{S}}}{R_{\mathrm{Ch}}} \tag{9}$$

While this relationship may lack quantitative accuracy owing to the approximations that were employed to arrive at the final equation, the general correlation between the normalized series resistance and fill factor modification holds true for moderate values of series resistance; and most importantly for the analysis contained in the main paper, a constant normalized series resistance has a less bandgap-dependent effect on photodiode fill factor than a constant value of series resistance, and is therefore more useful as a device quality parameter in our analysis.

3.2 Shunt resistance

For moderate values of shunt resistance, R_{Sh}, in an otherwise ideal diode, the modified maximum power point, P_{MP}', can be approximated as the ideal maximum power point, $P_{\mathrm{MP,ideal}}$, minus the shunt resistance power loss:

$$P_{\mathrm{MP}}^{'} = P_{\mathrm{MP,ideal}} - \frac{V_{\mathrm{MP}}^{2}}{R_{\mathrm{Sh}}} = P_{\mathrm{MP,ideal}}\left(1-\frac{V_{\mathrm{MP,ideal}}}{I_{\mathrm{MP,ideal}}}\frac{1}{R_{\mathrm{Sh}}}\right) \tag{10}$$

Using the same approximation for characteristic resistance as before (Eqn. 5) in conjunction with the assumption that open circuit voltage and short circuit current are unaffected by the resistance term, we

arrive at a relation between the modified fill factor, the ideal fill factor and the normalized shunt resistance, r_{Sh},

$$P'_{MP} = P_{MP,ideal}\left(1-\frac{R_{Ch}}{R_{Sh}}\right) = P_{MP,ideal}\left(1-r_{Sh}\right) \tag{11}$$

$$V'_{OC}\, j'_{SC}\, ff' = V_{OC,ideal}\, j_{SC,ideal}\, ff_{ideal}\left(1-\frac{1}{r_{Sh}}\right) \tag{12}$$

$$ff' = ff_{ideal}\left(1-\frac{1}{r_{Sh}}\right) \tag{13}$$

where the normalized shunt resistance, r_{Sh}, is defined as

$$r_{Sh} = \frac{R_{Sh}}{R_{Ch}} \tag{14}$$

While this relationship may lack quantitative accuracy owing to the approximations that were employed to arrive at the final equation, the general correlation between the normalized shunt resistance and fill factor modification holds true for moderate values of series resistance; and most importantly for the analysis contained in the main paper, a constant normalized shunt resistance has a more bandgap-independent effect on photodiode fill factor than a constant value of shunt resistance, and is therefore more useful as a device quality parameter in our analysis.

Supplementary References

1. Honsberg, C. B., Stuart Photovoltaic Education Network. www.pveducation.org.
